# A correlation study of preoperative lumbar paraspinal muscle quality and L5-S1 lumbar foraminal stenosis degeneration after L4–5 TLIF

**DOI:** 10.1186/s13018-023-04196-4

**Published:** 2023-09-27

**Authors:** Minghang Chen, Peng Zhang, Jiaxin Lai, Sheng Li, Weijie Yu, Shikang Fan, Honglin Teng

**Affiliations:** Department of Spine Surgery, The First Affiliated Hospital of Wenzhou Medical University, Wenzhou, 325000 Zhejiang Province China

**Keywords:** Lumbar foraminal stenosis, Lumbar fusion surgery, Muscle fatty infiltration, Muscle cross-section area, Adjacent segment degeneration, TLIF

## Abstract

**Study design:**

This was a retrospective study.

**Objectives:**

Adjacent segment degeneration (ASD) is a major complication associated with spinal fusion. The lumbar paraspinal muscle is an essential factor influencing the occurrence of ASD. This study aimed to investigate the effect of preoperative lumbar paraspinal muscle quality on L5-S1 adjacent lumbar foraminal stenosis degeneration (ASLFSD) after L4–5 transforaminal lumbar interbody fusion (TLIF).

**Methods:**

A total of 113 patients diagnosed with lumbar spinal stenosis at L4–5 were treated with TLIF. Lumbar paraspinal muscle measurements were obtained preoperatively and bilaterally from axial T2-weighted MR images. The measurements included the total cross-sectional area of psoas (PS-tCSA), of erector spinae (ES-tCSA), and of multifidus (MF-tCSA); and fatty infiltration of psoas (PS-FI), of erector spinae (ES-FI), and of multifidus (MF-FI). Foraminal measurements, including posterior disc height (PDH), disc-to-facet distance (D–F), foraminal height (FH), and foraminal area (FA), were obtained bilaterally using a computed tomography system. The association between lumbar paraspinal muscle quality and changes in foraminal measurements was also studied.

**Results:**

We observed that the FH and FA significantly reduced at 1 year postoperatively at the mean follow-up period of 41.56 ± 8.38 months (range, 43–50 months), and PDH, D–F, FH, and FA all significantly reduced at final follow-up. These changes in foraminal measurements were significantly and negatively correlated with PS-FI, ES-FI, and MF-FI.

**Conclusion:**

During the clinical follow-up, we found that patients with a higher degree of paraspinal muscle FI were more likely to develop L5-S1 ASLFSD after L4–5 TLIF.

## Introduction

Lumbar spinal stenosis (LSS) is a common degenerative spinal disease in the older population. After a detailed report on transforaminal lumbar interbody fusion (TLIF) surgery by Harms et al. [1] in 1998, TLIF became the major surgical treatment for LSS. Cole and McCall [1] reported that TLIF is minimally invasive, has less structural exposure, and minimises lamina, facet, and pars dissection compared with posterior lumbar interbody fusion (PLIF). Adjacent segment degeneration (ASD) is a major concern after fusion surgery. However, few studies have discussed ASD of the lumbar foramen [2–5].

The pathology of lumbar foraminal stenosis was first reported in 1927 [6]. Lumbar foraminal stenosis might have been caused by posterolateral osteophytes, herniated discs, laterally bulging annulus fibrosus, subluxation of the facet, and hypertrophic ligamentum [7]. The concept of foraminal stenosis was defined as lateral spinal stenosis [6]. Notably, the reconstructed sagittal images provide better visualisation of the foramen. The L5-S1 foramen, because of its anatomical and functional features and lumbosacral junction, is more susceptible to significant loading from the trunk and tends to have a higher incidence of degeneration [7].

The lumbar paraspinal muscle plays a vital role in the stability of the entire spine and the effectiveness of spine surgery. Muscle quality can be evaluated using the total cross-sectional area (tCSA) and fatty infiltration (FI). The previous studies have reported that patients with a lower CSA and higher muscle FI are more likely to have low back pain (LBP), ASD, facet joint arthropathy, and spinal misalignment [8–13].

To our knowledge, the correlation between paraspinal muscle quality and adjacent segment lumbar foraminal stenosis degeneration (ASLFSD) has not been previously investigated. Consequently, this study aimed to investigate the effects of preoperative paraspinal muscle tCSA and FI on L5-S1 ASLFSD after L4–L5 TLIF.

## Patients and methods

### Inclusion and exclusion criteria of participants

All participants met the following inclusion criteria: (1) conservative treatment failure after a minimum of 3 months, (2) age ≥ 40 years, and (3) single-level TLIF surgery at L4–L5. The following were the exclusion criteria: (1) any patient with body mass index (BMI) ≥ 30 kg/m^2^, (2) age < 40 years, (3) multilevel fusion surgery, (4) abnormal muscle activity or ambulation due to parkinsonism or neuromuscular disease, and (5) lumbar spondylolisthesis, lumbar isthmic spondylolysis, spine scoliosis, lumbosacral transitional vertebrae, and lumbar intervertebral instability in L5-S1 (dynamic segment angle change > 5°). Ultimately, 113 patients (54 males and 59 females) diagnosed with L4–5 LSS who underwent single-segment TLIF in our hospital between January 2018 and October 2021 were included in our study.

### Surgical technique

All the patients were placed in the prone position. The segments were located preoperatively using C-arm radiography. Lateral and anteroposterior images were obtained before surgery to determine the pedicle position of the surgical segment. Additionally, a posterior median incision was made, and the natural cleavage plane between the multifidus and longissimus muscles was separated to expose the facet joints bilaterally (Wiltse approach). After identification of the traversing and exiting nerve roots, an aggressive full discectomy was performed in Kambin’s triangle [14]. An appropriate height cage (Medtronic Sofamor Danek, Memphis, USA) filled with bone obtained from laminectomy, bone morphogenetic protein (rhBMP-2,4 mg, Hangzhou Jiuyuan, China) was inserted into the intervertebral space, and pedicle screws and a rob system were implanted. Notably, artificial bone or ilium was not used in any patient. All surgeries were performed by a senior spine surgeon.

#### Sagittal measurements

We measured the patient's lumbar lordosis (LL), pelvic incidence (PI), pelvic tilt (PT), sacral slope (SS), sagittal vertical axis (SVA), and pelvic incidence–lumbar lordosis mismatch (PI–LL) on standing full-length lateral radiographs of the spine preoperatively.

#### Foraminal measurements

A 64-row multidetector computed tomography (CT) system (version 3.0; INFINITT Healthcare Co., Ltd., Seoul, South Korea; slice < 5 mm) was used for all patients preoperatively, 1 year postoperatively, and at the final postoperative follow-up.

**Fig. 1:**
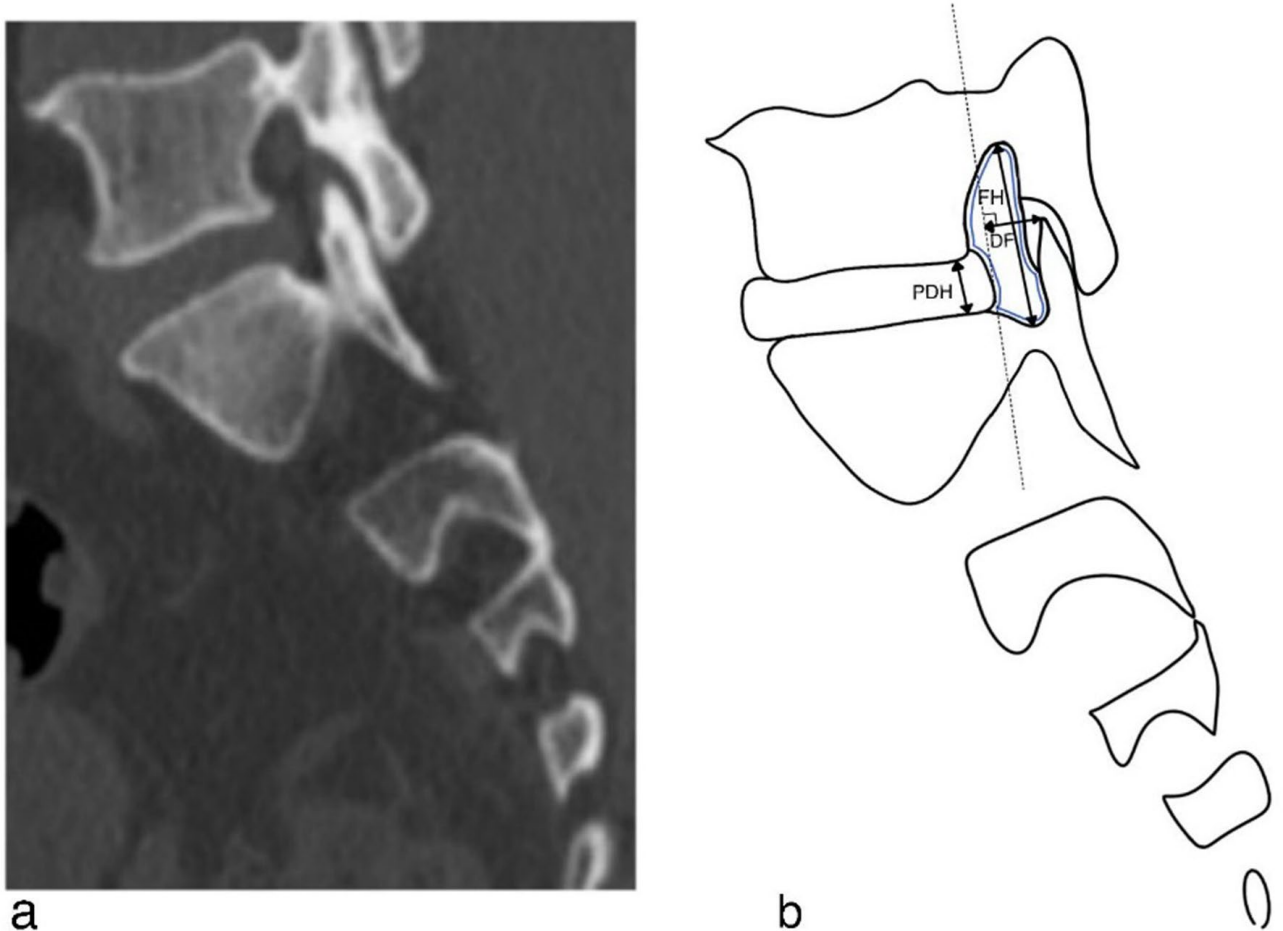
**a** The anatomical boundaries of L5-S1 foramen boundaries for CT scan in the sagittal plane. **b** The measurements made on the disc and intervertebral foramen. Posterior disc height (PDH): The distance between the upper and lower endplates of the involved disc. The disc-to-facet distance (D–F): The vertical distance between the apex of the superior articular process and the vertical line, defined as the caudal end of the bulging intervertebral disc to the inferior endplate in the sagittal plane. Foraminal height (FH): The maximum distance between the inferior margin of the pedicle of the superior vertebra and the superior margin of the pedicle of the inferior vertebra. Foraminal area (FA): FA is bounded by the surfaces of the upper and lower pedicles, the caudal end of the disc, and the anterior edge of the ligamentum flavum (the area circled by the blue line)

The anatomical boundaries of the foramen were composed of the adjacent superior–inferior vertebral pedicles, posteroinferior margin of the superior vertebral body, intervertebral disc, posterosuperior margin of the inferior vertebral body, ligamentum flavum, and facet joint as the posterior boundaries (Fig. 1a). We selected the level of the bilateral L5-S1 nerve root entrances to the foramen, which appears as the area between the medial edges of the superior and inferior pedicle cortical bone connections in the sagittal plane. Foraminal measurements included the posterior disc height (PDH, mm), disc-to-facet distance (D–F, mm), foraminal height (FH, mm), and foraminal area (FA, mm^2^) (Fig. 1b).

#### Lumbar paraspinal muscle measurements

**Fig. 2:**
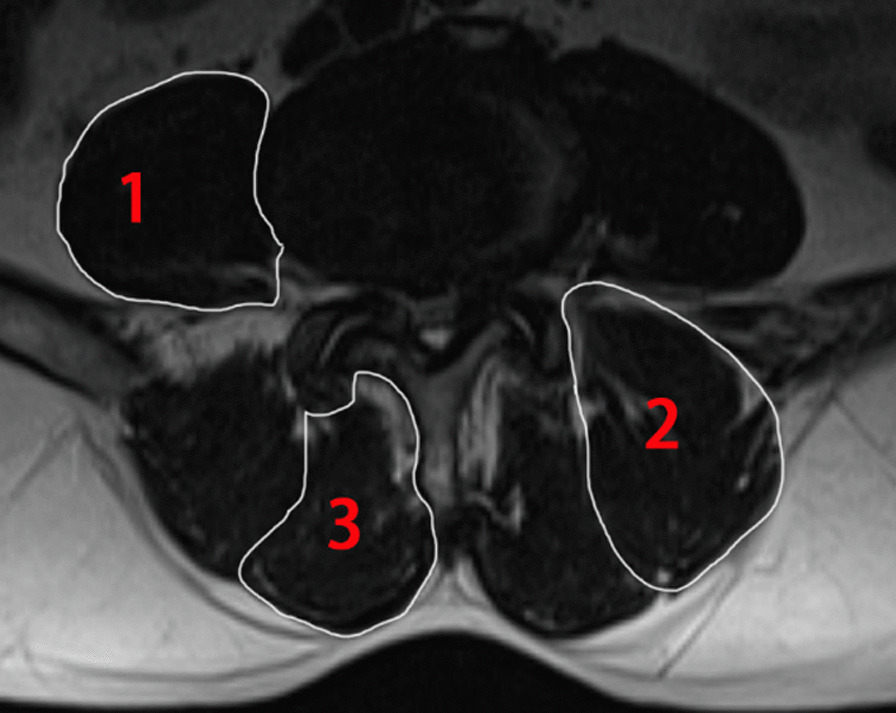
Region of interest (ROI) was used to measure the total cross-sectional area for the psoas, erector spinae, and multifidus. 1, psoas; 2, erector spinae muscle; and 3, multifidus

**Fig. 3:**
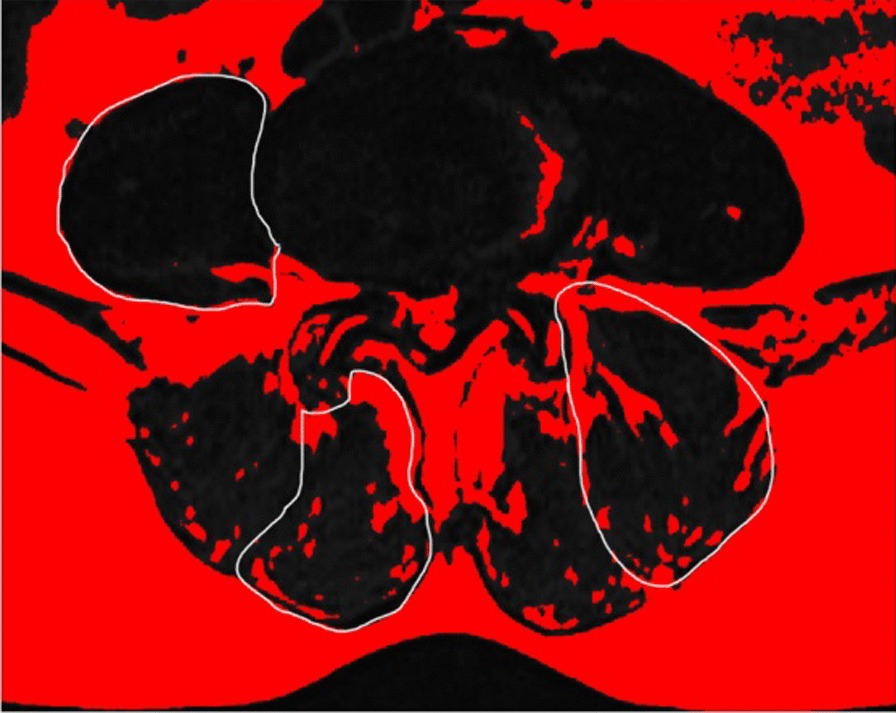
The thresholding technique was used to highlight the fatty tissue in the ROI muscle

Measurements of the lumbar paraspinal muscles were obtained from T2-weighted images using the ImageJ software. Magnetic resonance imaging (MRI) was conducted with a 1.5-T MRI superconducting imaging system (Siemens, Avanto, Germany)**.** Region of interest (ROI) was used in muscular measurements, including tCSA, in which we excluded the “tent”, which was defined as the region between the fascial plane and erector spinae [15, 16] (Fig. 2). The FI was defined as the area of fatty tissue measured using the thresholding technique (Fig. 3), and they reflect the quality of the lumbar paraspinal muscles.

All measurements were performed bilaterally at the inferior vertebral endplate of L4, including total cross-sectional area of psoas (PS-tCSA), of erector spinae (ES-tCSA), and of multifidus (MF-tCSA); and fatty infiltration of psoas (PS-FI), of erector spinae (ES-FI), and of multifidus (MF-FI). The tCSA of the muscle was standardised as the square of the patient’s height (cm^2^/m^2^).

#### CT-based classification system of LFS

**Fig. 4:**
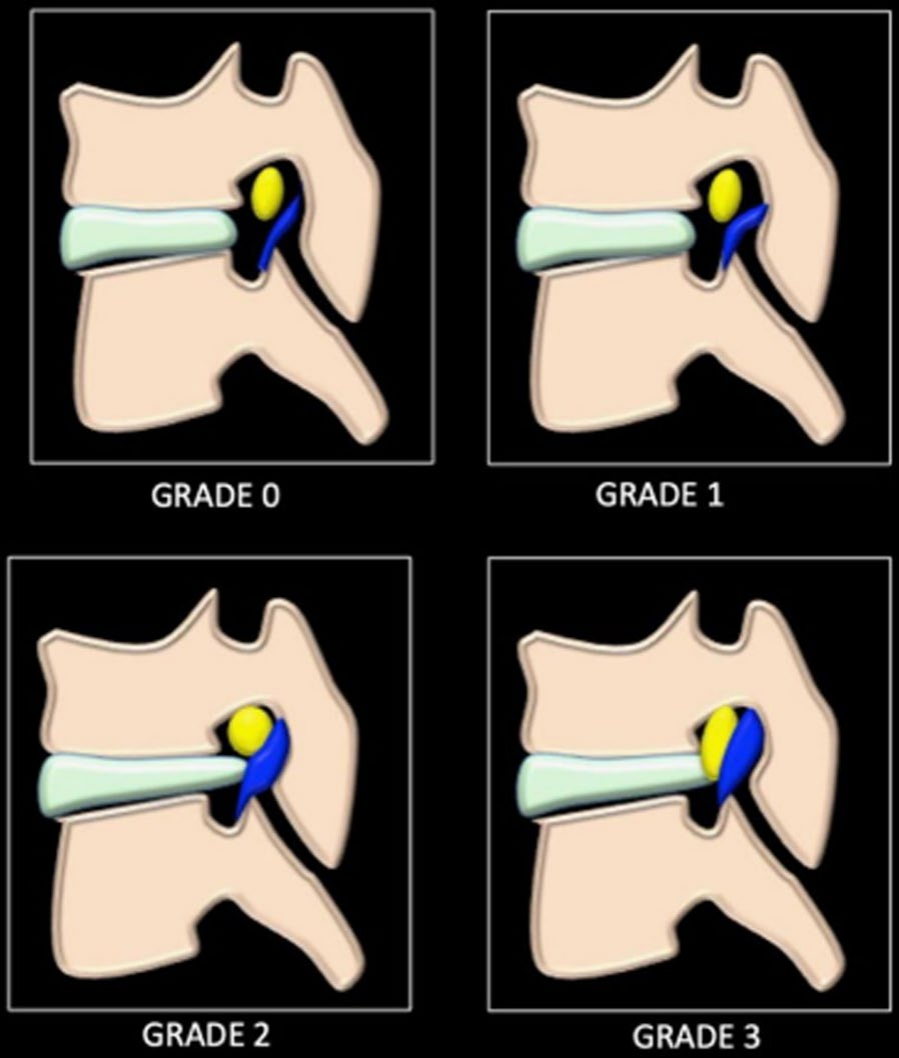
CT-based classification system of lumbar foraminal stenosis, normal foramen—grade 0, anteroposterior/superior–inferior fat compression—grade 1, both anteroposterior/superior–inferior compressions with no distortion of nerve root—grade 2, and grade 2 with additional distortion of nerve root—grade 3 [17]

Figure 4 shows the CT-based grading of lumbar foraminal stenosis (LFS) as proposed by Haleem et al. [17].

### Statistical analysis

Statistical analysis was performed using IBM SPSS Statistics 26 software (SPSS Inc., IBM Company Headquarters, Chicago, IL, USA). Correlations between the paraspinal muscle and lumbar foraminal measurements were computed using Pearson’s correlation analysis. An independent sample *t*-test and Chi-square test were performed to compare the differences between the groups. Statistical significance was set at *p* < 0.05. Notably, all parameters above were measured by an experienced orthopaedic surgeon.

## Results

### Patients characteristics

Table 1 summarises the basic characteristics of the included patients and lumbar paraspinal muscle measurements. Overall, 113 patients underwent L4–5 TLIF (right surgical approach: 54 and left surgical approach: 59), composed of 54 males and 59 females, and the mean follow-up period was 41.56 ± 8.38 months (range, 43–50 months). In total, 226 foramina were studied. The mean age of these patients was 62.49 ± 8.68 years, and the mean BMI was 24.49 ± 2.84 kg/m^2^.

**Table 1:** Patients’ characteristics

Variables	Value
Age (years)	62.49 ± 8.68
Number of patients/foramens	113/226
Surgical approach (right/left)	54/59
Gender (male/female)	54/59
BMI (kg/m^2^)	24.49 ± 2.84
Follow-up period (month)	41.56 ± 8.38
PS-tCSA(cm^2^/m^2^)	9.00 ± 2.23
ES-tCSA (cm^2^/m^2^)	10.90 ± 2.10
MF-tCSA (cm^2^/m^2^)	6.36 ± 0.98
PS-FI (%)	13.08 ± 7.06
ES-FI (%)	39.65 ± 14.55
MF-FI (%)	40.42 ± 16.79
PI	44.63 ± 7.91
PT	13.92 ± 5.13
SS	30.07 ± 8.59
LL	39.23 ± 9.99
PI–LL	5.40 ± 9.92
SVA	34.07 ± 22.19

BMI, body mass index; PS, psoas muscle; ES, erector spinae muscle; MF, multifidus muscle; tCSA, total cross-sectional area; and FI, fatty infiltration

### Foraminal measurements and correlations

The lumbar foramen measurements at 1 year postoperatively all reduced compared to those values preoperative, but only the FH (20.81 ± 2.71 and 20.31 ± 2.56, *p* < 0.05) and FA (63.00 ± 22.97 and 58.0 2 ± 20.41, *p* < 0.05) were significant. Compared with the preoperative values, PDH, FH, D–F, and FA were all significantly reduced at 1 year postoperatively, indicating the occurrence of L5-S1 foraminal stenosis after TLIF in the 1st year postoperatively (Tables 2 and 3).

**Table 2:** Foraminal measurements in L5-S1 before and after operation

		Foraminal measurements
		Mean ± SD
T1	PDH (mm)	3.90 ± 1.46
	D–F (mm)	5.49 ± 1.80
	FH (mm)	20.81 ± 2.71
	FA (mm^2^)	63.00 ± 22.97
T2	PDH (mm)	3.67 ± 1.50
	D–F (mm)	5.27 ± 1.65
	FH (mm)	20.31 ± 2.56^a^
	FA (mm^2^)	58.02 ± 20.41^a^
T3	PDH (mm)	3.31 ± 1.34^b^
	D–F (mm)	4.80 ± 1.51^b^
	FH (mm)	18.83 ± 2.61^b^
	FA (mm^2^)	50.87 ± 17.57^b^

PDH, posterior disc height; D–F, disc-to-facet distance; FH, foraminal height; FA, foraminal area; T1 = preoperatively; T2 = 1 year postoperatively; and T3 = final follow-up postoperatively

^a^Significant difference in preoperatively and 1 year postoperatively

^b^Significant difference in 1 month postoperatively and final follow-up postoperatively

**Table 3:** CT-based classification for lumbar foraminal stenosis in L5-S1 before and after operation

	Grade 0	Grade 1	Grade 2	Grade 3	Chi-square value	*p*
T1	31	45	113	37	16.311	0.012
T2	26	35	120	43		
T3	18	31	110	66		

T1 = preoperatively; T2 = 1 year postoperatively; and T3 = final follow-up postoperatively

*p* < 0.05 means significant correlation between grade and follow-up time

Table 4 shows the correlation between the lumbar foramen measurement change and the lumbar paraspinal muscle. Compared with preoperative measurements, changes in foramen measurements, including PDH, D–F, FH, and FA at the final postoperative follow-up, were positively correlated with preoperative PS-FI, ES-FI, and MF-FI. Therefore, the higher the muscle fat content, the more likely ASLFSD was to occur, whereas there was no significant correlation between preoperative paraspinal muscle tCSA and foramen measurement changes. Similarly, in the comparison of lumbar foramen measurements between the final follow-up and 1 year postoperatively, there was a positive correlation between D–F, FH, and FA changes and preoperative PS-FI, ES-FI, and MF-FI, while PDH did not show any significant correlation.

**Table 4:** Correlation between foraminal measurement data changes and muscle quality

		PS-tCSA (cm^2^/m^2^)	ES-tCSA (cm^2^/m^2^)	MF-tCSA (cm^2^/m^2^)	PS-FI (%)	ES-FI (%)	MF-FI (%)
T2 versus T3	ΔPDH (%)	− 0.072	− 0.016	0.092	0.161*	0.342*	0.364*
	ΔD–F (%)	− 0.082	− 0.046	0.058	0.202*	0.410*	0.385*
	ΔFH (%)	− 0.061	− 0.101	0.071	0.146*	0.232*	0.202*
	ΔFA (%)	− 0.058	0.032	0.053	0.175*	0.283*	0.298*
T1 versus T3	ΔPDH (%)	− 0.077	− 0.094	0.059	− 0.017	0.085	0.023
	ΔD–F (%)	− 0.113	− 0.071	0.014	0.209*	0.385*	0.284*
	ΔFH (%)	− 0.110	− 0.087	0.076	0.230*	0.372*	0.349*
	ΔFA (%)	− 0.089	− 0.103	0.065	0.146*	0.463*	0.496*

ΔPDH, change of posterior disc height; ΔD–F, change of disc-to-facet distance; ΔFH, change of foraminal height; and ΔFA, change of foraminal area

T1 = preoperatively; T2 = 1 year postoperatively; and T3 = final follow-up postoperatively

*p* < 0.05 is marked by “*” using Pearson correlation analysis

Table 5 shows that in the final follow-up postoperatively, according to the CT-based classification system of LFS, we compared paraspinal muscle quality between patients graded 3 and patients graded 0, 1, and 2. We found that there was a significant difference in the FI of the paraspinal muscle between the two groups of patients, and patients who with severe LFS have a higher degree of FI. However, interestingly, these patients also had a larger tCSA of paraspinal muscle, although no significant differences were observed.

**Table 5:** At final follow-up postoperatively, the difference of paraspinal muscle quality between group (grades 0, 1, and 2) and group (grade 3)

	Group (grades 0, 1, and 2, *n* = 74)	Group (grade 3, *n* = 39)
PS-tCSA (cm^2^/m^2^)	8.96 ± 2.05	9.07 ± 2.56
ES-tCSA (cm^2^/m^2^)	10.67 ± 2.07	11.34 ± 2.13
MF-tCSA (cm^2^/m^2^)	6.27 ± 1.05	6.52 ± 0.83
PS-FI (%)	12.14 ± 7.16	14.86 ± 6.58^a^
ES-FI (%)	36.27 ± 14.04	46.07 ± 13.45^a^
MF-FI (%)	35.88 ± 15.29	49.03 ± 16.31^a^

PS, psoas muscle; ES, erector spinae muscle; MF, multifidus muscle; tCSA, total cross-sectional area; and FI, fatty infiltration

^a^Significant difference between groups in paraspinal muscle measurements

## Discussion

ASD is common after lumbar fusion surgery, and adjacent foramen segment stenosis is often observed. Ryu et al. [18] reported that reoperation is most likely associated with foraminal stenosis in patients with ASD (*p* = 0.001). Our study aimed to investigate the relevance of preoperative paraspinal muscle quality in the occurrence of L5-S1 ASLFSD after L4–5 TLIF.

Changes in PDH, D–F, and FH could potentially lead to a reduction in FA owing to the anatomical structure of the intervertebral foramen. In our study, FH and FA were significantly reduced at 1 year postoperatively compared to preoperative foraminal measurements, and PDH, D–F, FH, and FA were all significantly reduced at the final postoperative follow-up compared to 1 year postoperatively. Although our study follow-up period was short, foraminal stenosis did occur after surgery. The reasons for the occurrence of L5-S1 ASLFSD after L4–5 TLIF also varied. The previous studies have shown that fusion surgery increases pressure in the intervertebral disc and facet joint in adjacent segments [1, 19–23]. The increase in biomechanical pressure promotes disc degeneration, further disc herniation, extrusion of the lumbar foramen, and a decrease in foraminal height [20, 24–26]. The accelerated degeneration of facet joints after fusion surgery may be another contributing factor to the change in foramen morphology [9–12].

Moreover, correlations between foraminal parameter changes, paraspinal muscle tCSA, and FI were analysed. Regardless of whether it was 1 year or the final postoperative follow-up, the FI of the paraspinal muscles was negatively correlated with changes in the foraminal measurements, though there was no significant correlation with PDH. Our results indicate that the tCSA of the paraspinal muscle is not a decisive factor affecting the degeneration of the intervertebral foramen and that the degree of muscular FI is a risk factor for the occurrence of ASLFSD. To further validate our hypothesis, we compared the difference in paraspinal muscle quality between patients with severe spinal stenosis (grade 3, based on the CT classification system of LFS) and general patients (grades 0, 1, and 2, based on the CT classification system of LFS) during the final follow-up. The patients of the grade 3 have higher degree of paraspinal muscle FI. However, we also found that tCSA of the paraspinal muscle was larger in these patients. These increases in tCSA have not brought improvement to the patients. So, we believe that muscular FI is the more valuable for predicting L5-S1 ASLFSD after L4–5 TLIF. Then, how do the paraspinal muscles work?

Paraspinal muscle quality influences surgical efficacy. The previous studies have reported that a smaller tCSA is associated with a poorer fusion rate in patients undergoing PLIF [27, 28]. Wang et al. [29] demonstrated that a smaller multifidus tCSA and higher multifidus FI on preoperative MRI scans were significantly associated with higher ODI scores both preoperatively and postoperatively. In the lumbar muscle system, the psoas attached directly to the vertebral bodies anterolaterally acts as the primary flexor muscle group, whereas the multifidus and erector spinae act as strong extensor muscle groups [19]. Additionally, McGill et al. [30] showed that the erector spinae reduce the compression force from 20 to 35% in a body experiment under external compression. When the multifidus was studied as an individual muscle, it acted more as a segmental stabiliser to enable the separate control of individual vertebrae [31]. Electromyography studies have confirmed this result and revealed that the multifidus plays a role in controlling intersegmental motion [32, 33]. Thus, we strongly believe that, with a higher paraspinal muscle, the FI was more likely to develop ASLFSD after fusion surgery.

Why did we choose the L5-S1 level as our research subject? Regarding anatomical factors, the L5-S1 disc is at the lowermost part of the spine and is the most variable area of lumbar spine activity. The disc of L5-S1 is also more prone to degeneration in lumbar fusion and LBP patients [34, 35]. However, the presence of preoperative disc degeneration did not show a significant correlation with the development of postoperative ASD [36]. A study on the degenerative stenosis of the L3–4 intervertebral foramen after L4–5 TLIF surgery can be further investigated.

This study has some limitations, including a relatively small sample size and short follow-up period. Furthermore, this study did not include the intervertebral foramen of L3–4. Undoubtedly, with longer follow-up times, the incidence of ASLFSD following TLIF surgery will increase, and with a larger sample size, the association between ASLFSD and paraspinal muscle quality will become more apparent. Therefore, a long-term and large-scale study can be extended in future. Additionally, since we only considered preoperative MRI appearance of the paraspinal muscles, postoperative muscle atrophy and fatty infiltration of the patients were not further discussed. Finally, spinal sagittal balance is another influencing factor that cannot be ignored, we will further verify its relationship with ASLFSD in our subsequent research.

Nevertheless, this study has several strengths. All surgical operations were performed in the natural cleavage plane between the multifidus and longissimus muscles to minimise the damage to the muscle [37]. This approach also has the advantages of less blood loss, fewer ASD rates, less damage to paraspinal muscle, and fewer additional surgical procedures [38, 39]. Moreover, our measurements of the foramen area were comprehensive, including foraminal height and width, which could help us understand ASLFSD in a three-dimensional way. Additionally, this study was the first to evaluate spinal muscle quality as a prognosticator of ASLFSD after TLIF surgery; thus, this study could be a cornerstone for further studies analysing the factors influencing postoperative radiological foraminal stenosis in fusion surgery.

In the previous studies, research on ASLFSD after lumbar fusion surgery was scarce. Our research can make that clinical physicians have a deeper understanding of ASLFSD and pay more attention to this issue, and provide some theoretical basis for future research.

## Conclusion

In our clinical follow-up, we found that patients with a higher degree of paraspinal muscle FI were more likely to develop L5-S ASLFSD after L4–5 TLIF.

## Data Availability

The datasets used and/or analysed during the current study are available from the corresponding author on reasonable request.

## Abbreviations

LSS: Lumbar spinal stenosis
ASD: Adjacent segment degeneration
TLIF: Transforaminal lumbar interbody fusion
PLIF: Posterior lumbar interbody fusion
tCSA: Total cross-sectional area
FI: Fatty infiltration
ASLFSD: Adjacent segment lumbar foraminal stenosis degeneration
PS-tCSA: Psoas total cross-sectional area
ES-tCSA: Erector spinae total cross-sectional area
MF-tCSA: Multifidus total cross-sectional area
PS-FI: Psoas fatty infiltration rate
ES-FI: Erector spinae fatty infiltration rate
MF-FI: Multifidus fatty infiltration rate
PDH: Posterior disc height
D–F: Disc-to-facet distance
FH: Foraminal height
FA: Foraminal area
ROI: Region of interest
LFS: Lumbar foraminal stenosis
LL: Lumbar lordosis
PI: Pelvic incidence
PT: Pelvic tilt
SS: Sacral slope
SVA: Sagittal vertical axis
PI–LL: Pelvic incidence–lumbar lordosis mismatch
